# Losing a non-native, gaining an endemic: Description of Camponotus (Myrmentoma) limbouros sp. nov. (Hymenoptera, Formicidae) from Cyprus

**DOI:** 10.3897/zookeys.1294.205360

**Published:** 2026-10-06

**Authors:** Jakovos Demetriou, Bonnie B. Blaimer, Christos Georgiadis, Jody H. Voges, Benoit Guénard, Lech Borowiec, Sebastian Salata

**Affiliations:** 1 Laboratory of Vector Ecology and Applied Entomology, Joint Services Health Unit Cyprus, BFC RAF Akrotiri BFPO 57, Akrotiri, Cyprus Laboratory of Vector Ecology and Applied Entomology, Joint Services Health Unit Cyprus Akrotiri Cyprus; 2 Enalia Physis Environmental Research Centre, Eleftherias 1, Xylotymvou, 7510, Larnaca, Cyprus Enalia Physis Environmental Research Centre Larnaca Cyprus; 3 Department of Ecology and Systematics, Faculty of Biology, National and Kapodistrian University of Athens, 15784 Athens, Greece Department of Ecology and Systematics, Faculty of Biology, National and Kapodistrian University of Athens Athens Greece; 4 Museum für Naturkunde, Leibniz Institute for Evolution and Biodiversity Science, Center for Integrative Biodiversity Discovery, Berlin, Germany Museum für Naturkunde, Leibniz Institute for Evolution and Biodiversity Science, Center for Integrative Biodiversity Discovery Berlin Germany; 5 Section of Zoology – Marine Biology, Department of Biology, Faculty of Science, National and Kapodistrian University of Athens, 15784 Athens, Greece Section of Zoology – Marine Biology, Department of Biology, Faculty of Science, National and Kapodistrian University of Athens Athens Greece; 6 Zoology Museum, Department of Biology, Faculty of Science, National and Kapodistrian University of Athens, 15784 Athens, Greece Zoology Museum, Department of Biology, Faculty of Science, National and Kapodistrian University of Athens Athens Greece; 7 Faculty of Life Sciences, Department of Biology, Humboldt Universität zu Berlin, Berlin, Germany Faculty of Life Sciences, Department of Biology, Humboldt Universität zu Berlin Berlin Germany; 8 School of Biological Sciences, The University of Hong Kong, Hong Kong SAR, China School of Biological Sciences, The University of Hong Kong Hong Kong China; 9 University of Wrocław, Department of Biodiversity and Evolutionary Taxonomy, Myrmecological Laboratory, Przybyszewskiego 65, 51-148 Wrocław, Poland University of Wrocław, Department of Biodiversity and Evolutionary Taxonomy, Myrmecological Laboratory Wrocław Poland

**Keywords:** Ants, *
Camponotus
*, DNA barcoding, endemic species, Mediterranean, taxonomy

## Abstract

The ever-growing movement of people and goods across the world has resulted in the translocation of tens of thousands of species outside their native ranges, including more than 500 species of ants. However, the native ranges of some non-native species are still unclear and further research using molecular tools is needed. Situated in the Eastern Mediterranean, Cyprus hosts a high percentage of both endemic and non-native ant taxa, with the latter species posing a threat to this native biodiversity, society, and the economy. The lack of up-to-date taxonomic revisions for many genera and knowledge gaps regarding the myrmecofauna of Western Asia and the Middle East pose challenges for the identification of some taxa, including a previously reported morphospecies labelled *Camponotus cf. vitiosus* from Cyprus and flagged as a potential non-native species. Here, we morphologically and molecularly (using COI barcoding) examine specimens of *C. cf. vitiosus*, comparing them with specimens of the nominal species from its native range as well as other *Camponotus* spp. Our re-examination reveals a new endemic species Camponotus (Myrmentoma) limbouros**sp. nov**., showing morphological and genetic affinities with the subgenus *Myrmentoma* Forel, 1912 in our COI barcode tree.

## Introduction

Invasive non-native species are a major driver of global biodiversity loss, affecting both people and nature (IPBES 2023). Ever-growing international commerce and transportation of people across the globe have led to increased species translocations to new regions outside their native range that continue without saturation (Hulme 2009; Seebens et al. 2017; Seebens 2019). As such, early warning, rapid detection and response are crucial steps to assess and manage biological invasions. With around 520 species transported around the world (Wong et al. 2023), ants constitute an important organismic group in invasion biology due to their severe impacts to native biodiversity, human health and the economy (Angulo et al. 2022; Gruber et al. 2022).

Within the last few years, several novel non-native ant species have been introduced to and established viable self-sustaining populations in Europe and the Mediterranean e.g., the Red Imported Fire Ant *Solenopsis invicta* Buren, 1972 (Menchetti et al. 2023), the Asian Needle Ant *Brachyponera chinensis* (Emery, 1895) (Menchetti et al. 2022), *Pheidole parva* Mayr, 1865 (Demetriou et al. 2025a; Karaman and Kiran 2026; Schifani and Massaad 2026), *Pheidole rosae* Forel, 1901 (Espadaler and Vila 2025), *Lepisiota capensis* (Mayr, 1862) (Reyes-López et al. 2025), and *Strumigenys perplexa* (Smith, 1876) (Hamer et al. 2021). Nevertheless, the native ranges of some non-native species are still unclear, including but not limited to, *Anochetus ghilianii* Forel, 1915, *Technomyrmex vexatus* (Santschi, 1919; Guillem and Bensusan 2008, 2019; Tinaut et al. 2011; Jowers et al. 2015; Arcos et al. 2026), *Cardiocondyla mauritanica* Forel, 1890 (Schifani 2019), *Monomorium bicolor* Emery, 1877 (Demetriou et al. 2023a, 2023b) and *Hypoponera punctatissima* (Roger, 1859; Seifert 2003). Such cases could be further researched using molecular tools such as the DNA Barcode Reference Library for European Ants (Menchetti et al. 2026), constituting an important step towards deciphering species’ taxonomy and distribution patterns.

The island of Cyprus is situated in the heart of the Eastern Mediterranean, bordered by Europe, Asia and Africa. Latest published studies on its myrmecofauna synthesize a checklist of 88 species, including 18 endemic and 18 non-native taxa (each with 20.5% of all species) (Demetriou et al. 2025b). However, more taxa have been recently uncovered, with the island’s myrmecofauna steadily approaching 100 species (Demetriou et al. unpublished data). The high percentage of endemic species highlights the importance of Cyprus as a biodiversity hotspot and rarity center for Mediterranean ants (Kass et al. 2022), but the large portion of non-native species is posing a threat to this native biodiversity, society and the economy (Demetriou et al. 2023b). In addition to the lack of up-to-date taxonomic revisions in general for many genera, the myrmecofauna of Western Asia and the Middle East is especially poorly known. Given the proximity of Cyprus to the region, this poses challenges for identification of some taxa, including representatives of the genera *Lepisiota*, *Solenopsis* and *Tetramorium* (see: Statistics and checklist in Demetriou et al. 2025b). Two taxa in recent studies had not been identifiable to the species level, i.e., *Camponotus cf. vitiosus* (Demetriou et al. 2023b; Salata et al. 2023) and *Solenopsis* sp_CYP139 (Demetriou et al. 2023b), and were flagged as potential non-native species, necessitating further investigations utilizing molecular tools and comparisons with specimens from their hypothesized native range.

Native to East Asia (China, Japan, Korean Peninsula), *Camponotus vitiosus* Smith, F., 1874 belongs to the subgenus *Myrmamblys* Forel, 1912, which holds no representatives in the Western Palearctic (Janicki et al. 2016; Guénard et al. 2017). The sole record of a potential introduction to the Mediterranean stems from Ionescu-Hirsch (2010 [2009]), reporting on specimens identified as “*Camponotus* (*Myrmamblys*) sp. near *vitiosus* Smith, F., 1874”, introduced to Israel through bamboo from China in two separate cases in 2006 and 2009. In 2012, one gyne and one minor worker of *Camponotus cf. vitiosus* were collected from Cyprus (Salata et al. 2023). Specimens were identified as members of the subgenus *Myrmamblys*, with *C. vitiosus* being the most probable candidate. This finding recently prompted a search for further individuals of the taxon as well as the use of molecular tools for its robust identification.

In this article, specimens of *C. cf. vitiosus* from Cyprus are morphologically and molecularly (DNA barcoding) compared with specimens of *C. vitiosus* from its native range as well as other *Camponotus* species from the Mediterranean region and beyond. Upon re-examination and comparisons, Cypriot specimens are found nested in the subgenus *Myrmentoma* Forel, 1912 and are herein described as a new endemic species for the island, Camponotus (Myrmentoma) limbouros sp. nov.

## Materials and methods

### Taxonomic and molecular examination

To examine the conspecificity of specimens identified as *Camponotus cf. vitiosus* from Cyprus with the nominal species *C. vitiosus*, we compared collected material of all castes from Cyprus sampled in 2012 by L. Borowiec and S. Salata and 2024 sampled by S. Salata, C. Georgiadis and J. Demetriou with material provided by B. Guénard from Hong Kong (China) and Japan.

We further assembled a genetic dataset of cytochrome oxidase I (COI) to investigate the identity of *C. cf. vitiosus* with molecular methods. For this, we generated COI barcode sequences for one *C. cf. vitiosus* sample from Cyprus and one *C. vitiosus* sample from Japan. To obtain a comprehensive dataset for genetic comparison and phylogenetic reconstruction, we downloaded 189 sequences from the Barcode of Life Data Systems (BOLD v. 5; Ratnasingham and Hebert 2007; https://boldsystems.org, accessed between 12–16 May 2026). This dataset comprised all Camponotus (Myrmentoma) species (≤ 10 specimens per species) for which sequence data were available, sequences from other *Camponotus* species and subgenera occurring in Cyprus and adjacent regions in the Eastern Mediterranean (≤ 5 specimens per species), as well as one sequence of *Colobopsis truncata* (Spinola, 1808) and two sequences of *Colobopsis minus* (Wang & Wu, 1994) as outer outgroups (Suppl. Table SS1). The latter have been downloaded from BOLD as Camponotus (Myrmentoma) minus, but this turned out to be outdated taxonomy post-analysis as the species was recently transferred to *Colobopsis* (Ye et al. 2025). We complemented this dataset with four additional samples generated by us for other Camponotus (Myrmentoma) species; thus including six COI barcodes generated at the Museum für Naturkunde, Berlin (ZMB) (Suppl. Table S2), following methodology outlined below. Table 1 summarizes the included species and specimen numbers. Newly generated sequences have been submitted to BOLD (project CAMY).

**Table 1.** Summary of *Camponotus* sequences included in the full molecular analysis, separated by source.

**Genus (subgenus) species**	**Authority**	**BOLD**	**This study**
Camponotus (Camponotus) herculeanus	(Linnaeus, 1758)	5	
Camponotus (Camponotus) ligniperda	(Latreille, 1802)	5	
Camponotus (Camponotus) schaefferi	Wheeler, W.M., 1909	2	
Camponotus (Camponotus) vagus	(Scopoli, 1763)	5	
Camponotus (Myrmamblys) vitiosus	Smith, F., 1874	4	1
Camponotus (Myrmentoma) aegaeus	Emery, 1915	5	
Camponotus (Myrmentoma) anthrax	Wheeler, W.M., 1911	1	
Camponotus (Myrmentoma) atricolor	(Nylander, 1849)	9	
Camponotus (Myrmentoma) bakeri	Wheeler, W.M., 1904	1	
Camponotus (Myrmentoma) boghossiani	Forel, 1911	3	
Camponotus (Myrmentoma) candiotes	Emery, 1894	1	1
Camponotus (Myrmentoma)* confucii	Forel, 1894	1	
Camponotus (Myrmentoma) cf. vitiosus (=limbouros)	sp. nov.		1
Camponotus (Myrmentoma) dalmaticus	(Nylander, 1849)	10	
Camponotus (Myrmentoma) essigi	Smith, M.R., 1923	2	
Camponotus (Myrmentoma) fallax	(Nylander, 1856)	10	
Camponotus (Myrmentoma) gestroi	Emery, 1878	9	
Camponotus (Myrmentoma) heidrunvogtae	Seifert, 2019	1	1
Camponotus (Myrmentoma)* himalayanus	Forel, 1893	1	
Camponotus (Myrmentoma) hyatti	Emery, 1893	10	
Camponotus (Myrmentoma) kiesenwetteri	(Roger, 1859)	5	
Camponotus (Myrmentoma) lateralis	(Olivier, 1792)	10	
Camponotus (Myrmentoma) nearcticus	Emery, 1893	10	
Camponotus (Myrmentoma) nitidescens	Forel, 1889	2	
Camponotus (Myrmentoma) piceus	(Leach, 1825)	9	
Camponotus (Myrmentoma)* quadrinotatus	Forel, 1886	2	
Camponotus (Myrmentoma) rebeccae	Forel, 1913	1	2
Camponotus (Myrmentoma) ruber	Emery, 1925	1	
Camponotus (Myrmentoma) sayi	Emery, 1893	5	
Camponotus (Myrmentoma) spissinodis	Forel, 1909	2	
Camponotus (Myrmentoma)* subbarbatus	Emery, 1893	1	
Camponotus (Myrmentoma)* tergestinus	Müller, 1921	3	
Camponotus (Tanaemyrmex) aethiops	(Latreille, 1798)	5	
Camponotus (Tanaemyrmex) baldaccii	Emery, 1908	3	
Camponotus (Tanaemyrmex) buddhae	Forel, 1892	1	
Camponotus (Tanaemyrmex) fellah	Dalla Torre, 1893	3	
Camponotus (Tanaemyrmex) ionius	Emery, 1920	5	
Camponotus (Tanaemyrmex) jaliensis	Dalla Torre, 1893	1	
Camponotus (Tanaemyrmex) laconicus	Emery, 1920	3	
Camponotus (Tanaemyrmex) oertzeni	Forel, 1889	5	
Camponotus (Tanaemyrmex) samius	Forel, 1889	5	
Camponotus (Tanaemyrmex) sanctus	Forel, 1904	5	
Camponotus (Tanaemyrmex) thoracicus	(Fabricius, 1804)	3	
Camponotus (Tanaemyrmex) turkestanus	André, 1882	1	
Camponotus (Tanaemyrmex) universitatis	Forel, 1890	5	
Camponotus (Tanaemyrmex) xerxes	Forel, 1904	5	
*Colobopsis truncata*	(Spinola, 1808)	1	
*Colobopsis minus*	(Wang & Wu, 1994)	2	

*subgeneric assignment to *Myrmentoma* likely incorrect based on this study and unpublished phylogenomic data (Blaimer, unpublished data).

Specimens were deposited at the Museum of Natural History, University of Wrocław, Poland (**MNHW**), the Museum of Zoology of the National and Kapodistrian University of Athens, Athens, Greece (**ZMUA**) and the Museum für Naturkunde, Berlin, Germany (**ZMB**).

### Generation of COI barcodes

Laboratory processing followed the NGS species discovery pipeline at ZMB and was based on established Oxford Nanopore Technologies amplicon barcoding workflows (Srivathsan et al. 2021; Vasilița et al. 2024). Briefly, DNA was extracted from individual specimens using a modified HotSHOT protocol with 65 °C for 1 h. The 658 bp barcode region of the mitochondrial cytochrome c oxidase subunit I (COI) gene was amplified by PCR using specifically designed, uniquely indexed primers, enabling specimen-specific assignment of the resulting amplicons after sequencing (Srivathsan et al. 2021; Vasilița et al. 2024). PCR products were pooled and prepared for Oxford Nanopore sequencing using a ligation-based amplicon library preparation approach. Sequencing was performed on a PromethION platform (Oxford Nanopore Technologies, Oxford, UK), and specimen-specific barcode sequences were recovered after demultiplexing and barcode consensus generation within the ZMB pipeline using ONTbarcoder 2.0 (Srivathsan et al. 2024). Only barcodes with up to 1% ambiguities and up to 5 errors were used for analysis. The resulting COI barcode sequences were compared against BOLD using BOLDigger3 v. 2.3.0 (Buchner and Leese 2020) to obtain sequence-based taxonomic identifications.

### Genetic analyses

All 195 sequences were combined into one fasta file and aligned using Muscle (Edgar 2022) in AliView 1.28 (Larsson 2014), followed by manual trimming of indels in some sequences (as potential sequencing errors) and edge trimming of the alignment to 658 bp length. Our own sequences were hereby used as reference as these had passed stringent filtering criteria. We verified that the final alignment contained no stop codons by visualizing its amino acid translation. We partitioned the sequence alignment into two subsets (1^st^ +2^nd^ vs 3^rd^ codon position) and performed a joint Maximum Likelihood best tree and bootstrap analysis in IQ-TREE v. 3.0.1 (Minh et al. 2020), employing ModelFinder (Kalyaanamoorthy et al. 2017) to select the best-fitting substitution models (settings: -m MFP -p 2part -wbtl -bb 1000 -alrt 1000 -bnni -nm 5000 -nt 12 -safe).

We used the same alignment to calculate genetic distances in MEGA12 (Stecher et al. 2020, 2025). We used the Kimura 2-parameter model (Kimura 1980) to estimate pairwise sequence divergence, including all nucleotide positions and applying the pairwise deletion option to all ambiguous positions for each sequence pair. We applied a hypothetical barcode gap of 5% to distinguish between intra- and interspecific variation (Menchetti et al. 2026).

Photographs were taken using a Nikon SMZ 1500 stereomicroscope, Nikon D5200 photo camera, and Helicon Focus software. Label data for type material of the new species are in the original spelling, presented in square brackets; a vertical bar (|) separates data on different rows and double vertical bars (||) separate labels. Maps were created in QGIS, a free, open-source Geographic Information System Version 3.44 (https://qgis.org/en/site/).

The pilosity inclination degree follows that used in Wilson (1955). Appressed (0–5°) hairs run parallel or nearly parallel to the body surface. Decumbent hairs stand 5–20°, subdecumbent hair stands 20–60°, suberect 60–80°, and erect 80–90° from the surface (Wilson 1955: fig. 3).

All observations and measurements were made using a pin-holding stage, permitting rotations around the X, Y, and Z axes at magnifications from 32× to 100× with a Nikon SMZ 1500 microscope and an orthogonal crosshair micrometer. All measurements are presented in millimeters (mm) as an arithmetic mean, with minimum and maximum values in parentheses.

**EL** eye length; maximum diameter of the compound eye;

**HFL** hind femur length; measured on dorsal side from trochanter to apex of femur;

**HL** head length; measured in a straight line from mid-point of anterior clypeal margin to mid-point of posterior margin in full-face view, excavations of hind vertex and / or clypeus, if any, reduce HL;

**HW** head width; measured in full-face view directly posterior of the compound eyes;

**MSW** in gynes only, mesoscutum width: maximum width of mesoscutum in dorsal view;

**PW** workers only, pronotum width: maximum width of pronotum in dorsal view;

**PTH** petiole height; the chord of ventral petiolar profile at node level is the reference line perpendicular to which the maximum height of petiole is measured, measured from petiolar sternum to apex in lateral view;

**PTW** petiole width; maximum width of the petiolar node in lateral view;

**SL** scape length; maximum straight-line length of scape excluding the basal condylar bulb;

**ML** mesosoma length; measured as diagonal length from the anterior end of the neck shield to the posterior margin of the propodeal lobe.

Indices:

**CI** cephalic index, HW/HL × 100;

**SI** scape index, SL/HW × 100;

**PI** petiole index, PTH/PTW × 100;

**FI** femur index, HFL/ML × 100.

Abbreviations:

**w** worker;

**q** gyne;

**s** major worker.

## Results

### Molecular results

**Figure 1.**
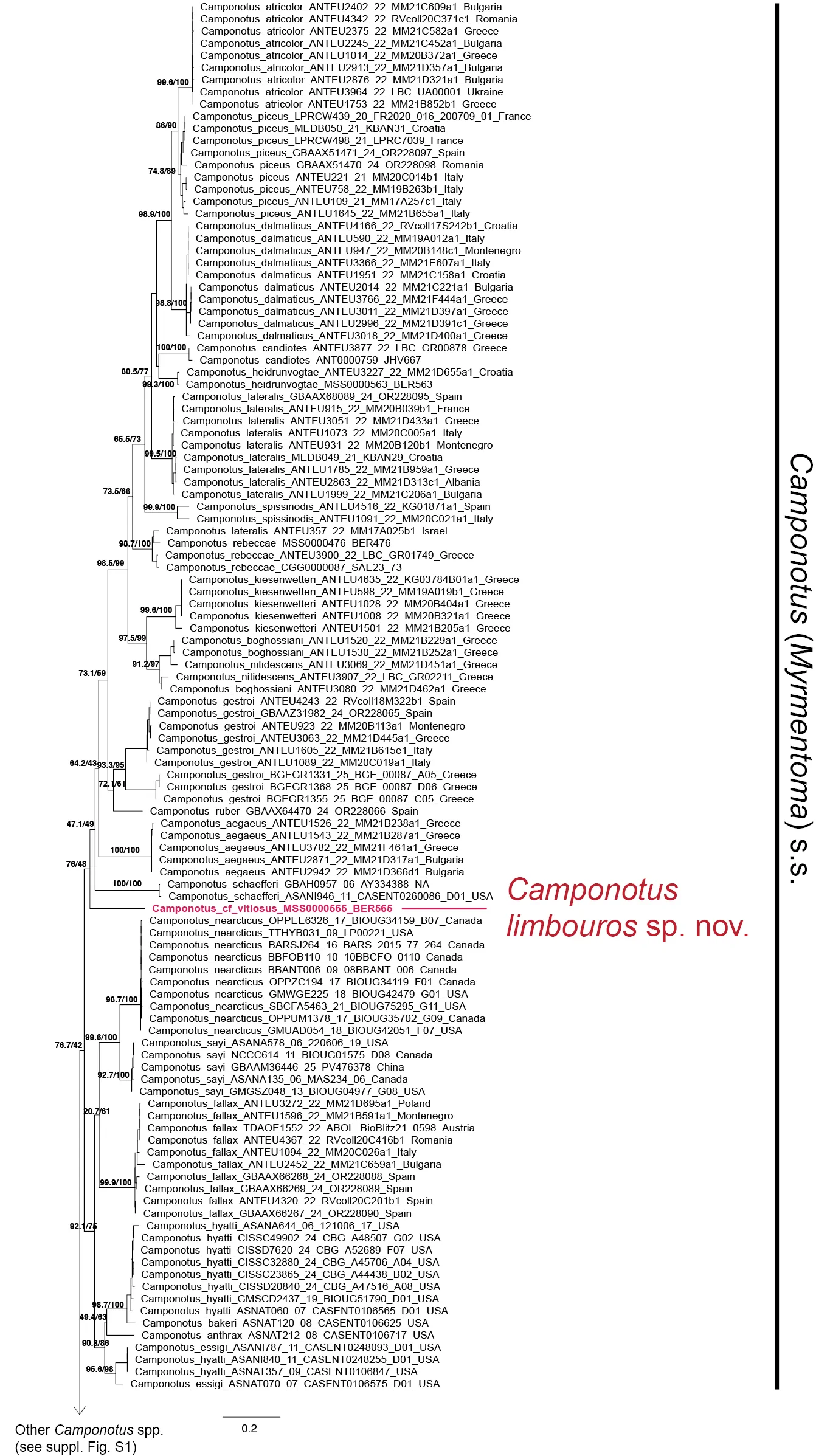
Cytochrome oxidase I (COI) tree for Camponotus (Myrmentoma). The tree was generated in a partitioned (1+2nd vs 3rd position) Maximum Likelihood best tree and bootstrap analysis using the 658 bp barcode region of COI and 195 taxa. The 195 taxa include all members of the subgenus *Myrmentoma* for which sequence data were available on BOLD v. 5 (https://boldsystems.org), as well as other *Camponotus* species which occur in Cyprus and the Eastern Mediterranean region, and the outgroup *Colobopsis* used for rooting. The comprehensive tree was pruned for display to include only the 114 sequences in the subgenus *Myrmentoma* (s.s.); a full version is available in the Suppl. material 1. Support values are SH-aLRT (left) and ultrafast bootstrap (right); values for intraspecific divergences have been removed to reduce cluttering. Scale bar represents nucleotide substitutions per site.

The COI data support *C. limbouros* sp. nov. as a distinct species. Genetic distances to other Camponotus (Myrmentoma) species range from 0.13 (*C. hyatti*) to 0.19 (*C. lateralis*, *C. kiesenwetteri*) base pair sequence divergence, and from 0.16 (*C. quadrinotatus*, *C. ligniperda*) to 0.24 (*C. xerxes*) to other *Camponotus* species included in the analysis (Supp. Table S3). Sequence divergence of *C. limbouros* sp. nov. to the nominal *C. vitiosus* is between 0.21–0.22. However, the phylogenetic analysis was not able to resolve the phylogenetic position of the species with certainty. The COI gene tree shows *C. limbouros* sp. nov. clustering within a clade of Palearctic and Nearctic Camponotus (Myrmentoma) species (here dubbed *Myrmentoma* s.s.), but the branch support for this grouping is low (SH-aLRT=76 and ultrafast bootstrap=48 for a sister relationship with Palearctic *Myrmentoma* s.s.) and the topology therefore unreliable (Fig. 1; Suppl. materials 1, 2–4). Likewise, support values across the remaining COI gene tree are moderate to poor on deeper phylogenetic levels (Fig. 1; Suppl. material 1), as expected from analysis of such a short mitochondrial fragment.

### Synopsis of members of the *Camponotus* subgenus *Myrmentoma* from the Eastern Mediterranean Region

*Camponotus fallax* group:

*Camponotus fallax* (Nylander, 1856);

*Camponotus tergestinus* Müller, 1921;

*Camponotus kiesenwetteri* group:

*Camponotus aegaeus* Emery, 1915;

*Camponotus aktaci* Karaman, 2013;

*Camponotus boghossiani* Forel, 1911;

*Camponotus kiesenwetteri* (Roger, 1859);

*Camponotus libanicus* André, 1881;

*Camponotus nitidescens* Forel, 1889;

*Camponotus schulzi* Salata, Loss, Karaman, Kiran & Borowiec, 2019;

*Camponotus lateralis* group:

*Camponotus abrahami* Forel, 1913;

*Camponotus anatolicus* Karaman & Aktac, 2013;

*Camponotus atricolor* (Nylander, 1849);

*Camponotus candiotes* Emery, 1894;

*Camponotus dalmaticus* (Nylander, 1849);

*Camponotus ebneri* Finzi, 1930;

*Camponotus gestroi* Emery, 1878;

*Camponotus heidrunvogtae* Seifert, 2019;

*Camponotus hirtus* Karaman & Aktac, 2013;

*Camponotus honaziensis* Karaman & Aktac, 2013;

*Camponotus interjectus* Mayr, 1877;

*Camponotus kurdistanicus* Emery, 1898;

*Camponotus lateralis* (Olivier, 1792);

*Camponotus piceus* (Leach, 1825);

*Camponotus rebeccae* Forel, 1913;

*Camponotus vogti* Forel, 1906;

*Incertae sedis*:

*Camponotus limbouros* sp. nov.

### Taxonomy

#### Camponotus limbouros sp. nov.

Taxon classificationAnimaliaHymenopteraFormicidae

46CC5AD3-5D63-59E3-8EC5-167A00D44D05

https://zoobank.org/5462CA76-B972-4A5D-A6EF-816F2E08DE46

Figs 2, 3, 4, 5, 6

= Camponotus cf. vitiosus: Salata et al. (2023): 9–13.
= Camponotus cf. vitiosus: Demetriou et al. (2023b): 24–26.

##### Type material examined.

**Figure 2.**
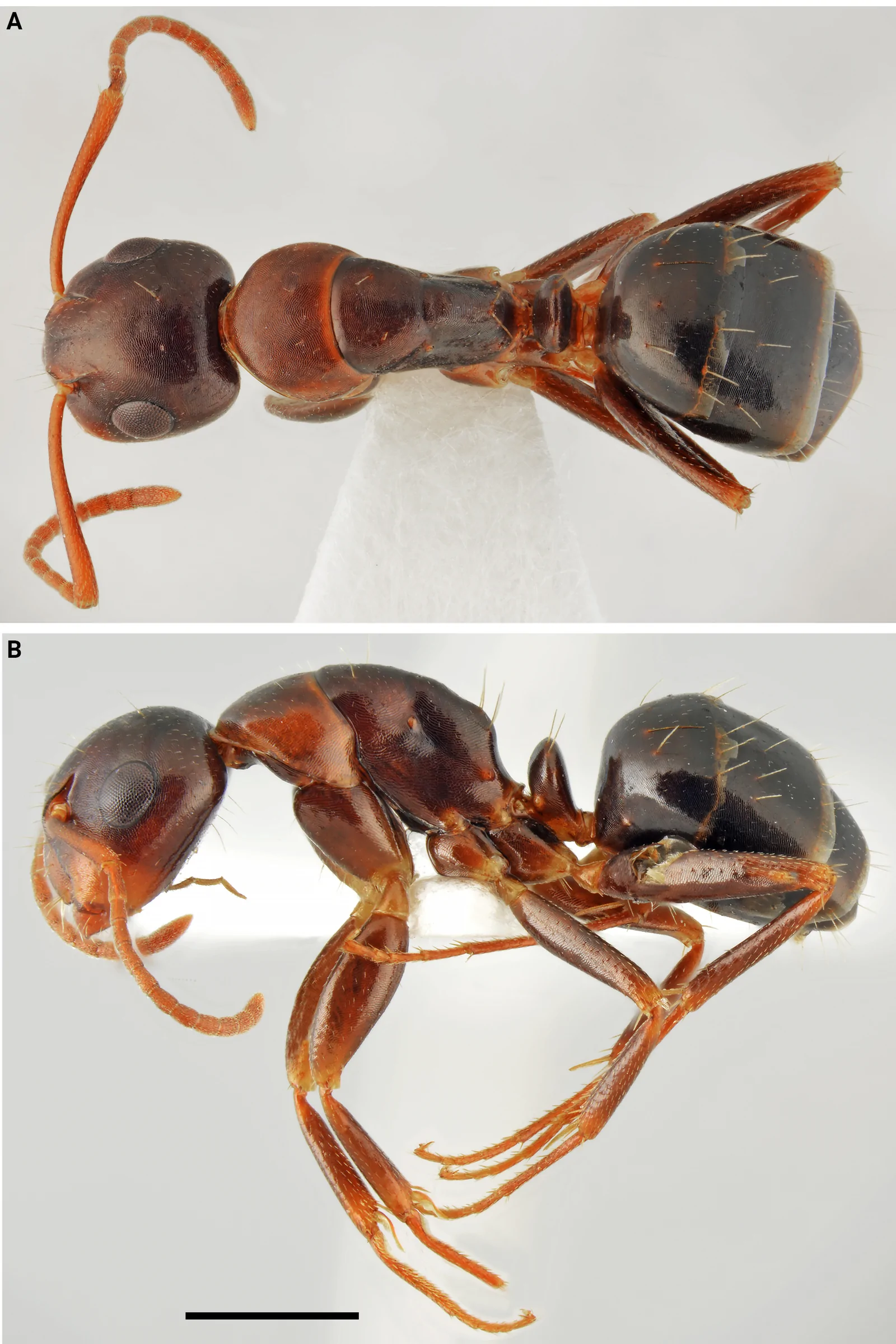
Minor worker (paratype) of *Camponotus limbouros* sp. nov. **A**. Dorsal view; **B**. Lateral view. Scale bar: 1 mm.

**Figure 3.**
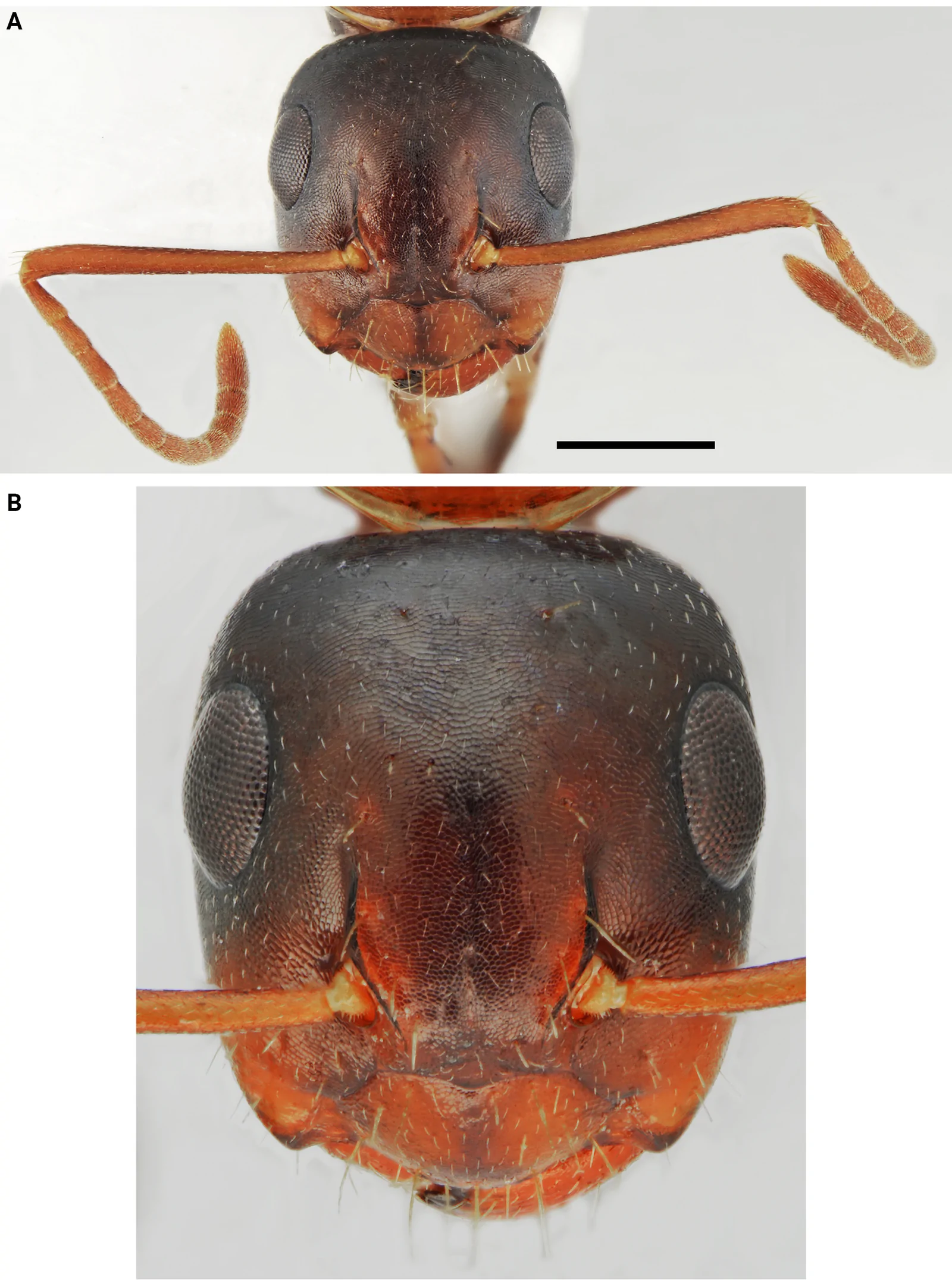
Minor worker (paratype) of *Camponotus limbouros* sp. nov. worker. **A**. Head and antennae; **B**. Worker head sculpture. Scale bar: 0.5 mm.

**Figure 4.**
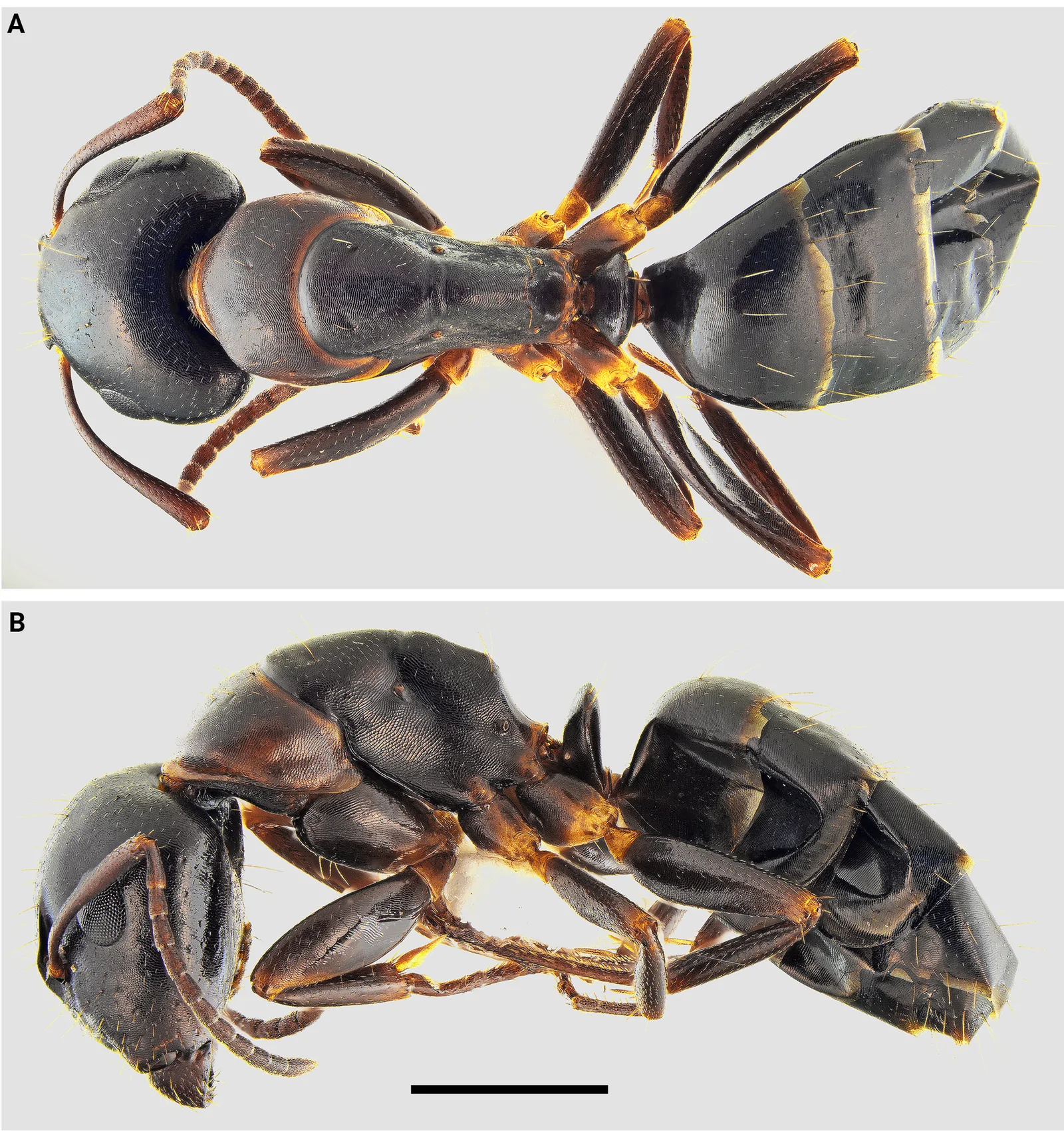
Major worker (holotype) of *Camponotus limbouros* sp. nov. **A**. Dorsal view; **B**. Lateral view. Scale bar: 1 mm.

**Figure 5.**
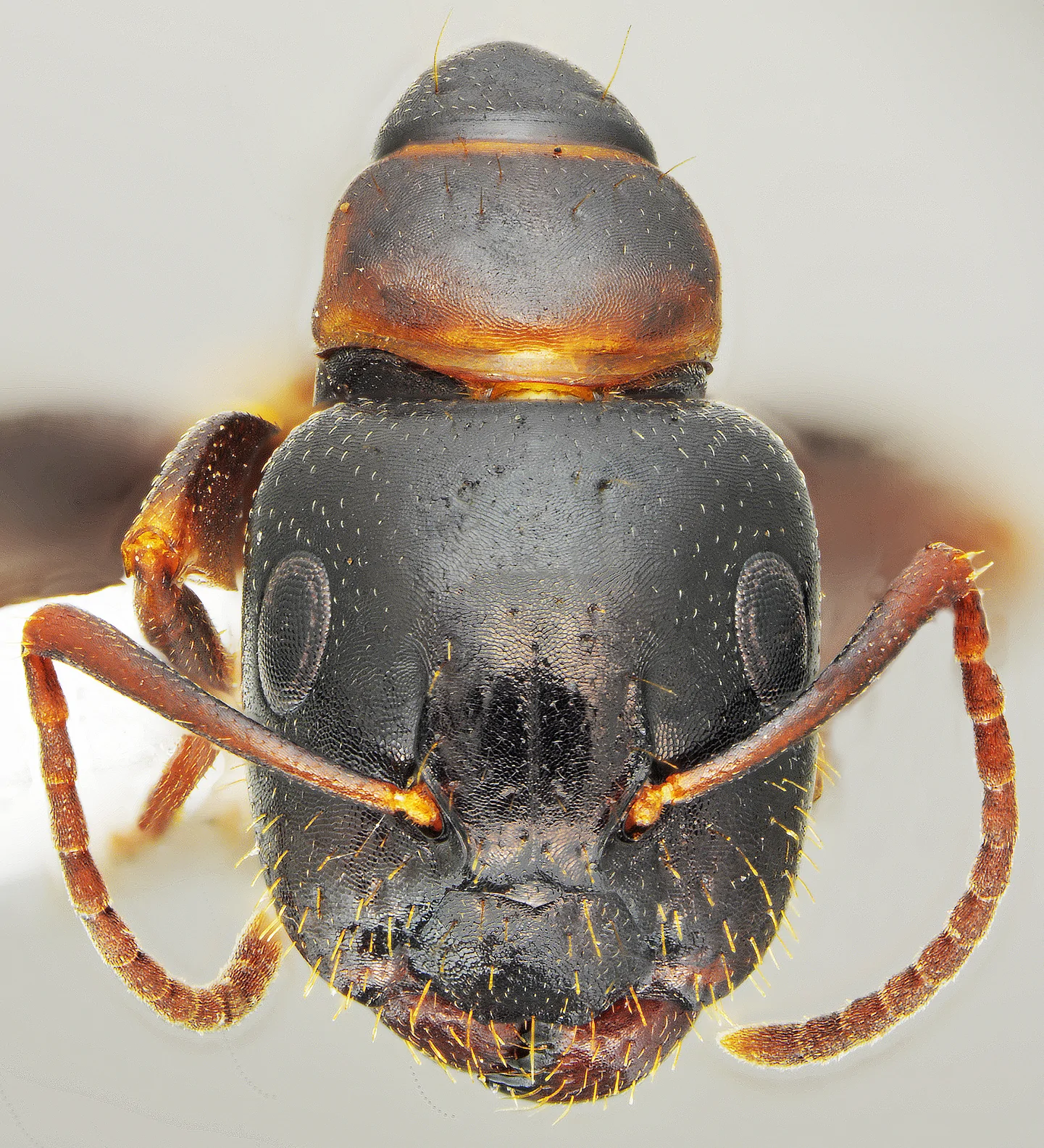
Major worker (holotype) of *Camponotus limbouros* sp. nov. Head and antennae. Scale bar: 0.5 mm.

**Figure 6.**
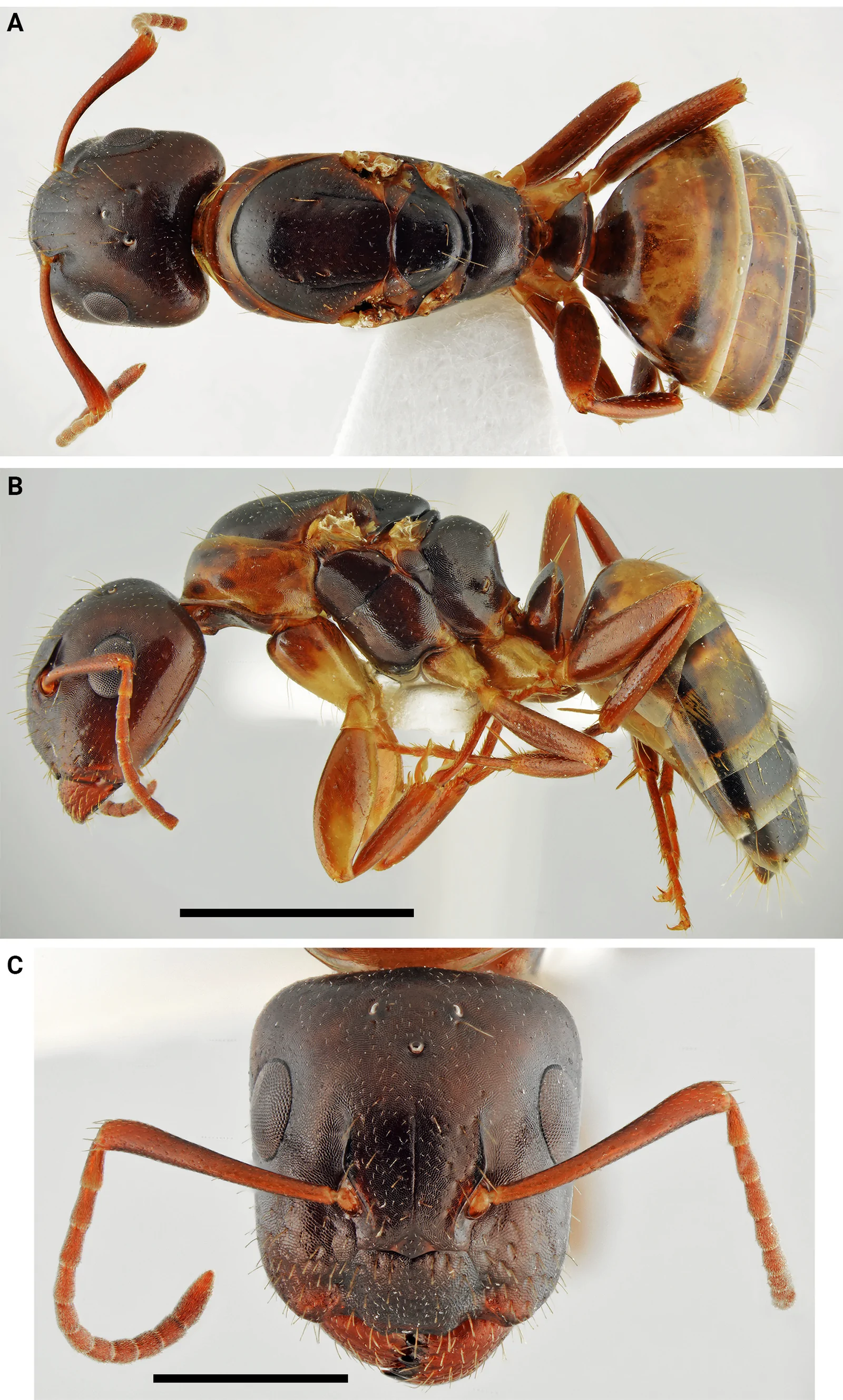
Gyne (paratype) of *Camponotus limbouros* sp. nov. **A**. Dorsal view; **B**. Lateral view; **C**. Head and antennae. Scale bars: 2 mm (**B**); 1 mm (**C**).

***Holotype***. • s. (pin), Cyprus, Paphos | Cedar Valley, 1130 m. | 34.9909 / 32.6883 | 10 VI 2024, leg. S. Salata (MNHW, DBETCV003) (Figs 4, 5). ***Paratypes***. • 2s., 10w., 1q.: the same data as holotype (MNHW, ZMUA, ZMB, DBETCV004-DBETCV016); • 1w., 1q.: Paphos, Cedar Valley, 34.98527, 32.68388, 1196 m a.s.l., 05.V.2012, Lech Borowiec leg. and det., (DBETCV001-DBETCV002) (MNHW) (Figs 2, 3, 6).

##### Other material examined.

• Lemessos, Pissouri, Trachonas 1, 34.6472, 32.6951, 225 m a.s.l., 21.VII–04.VIII.2013, Katerina Leonidou leg.; Jakovos Demetriou, Sebastian Salata, and Lech Borowiec det.; pitfall traps, 300 m a.s.l., phrygana with *Ceratonia siliqua*; ZMUA, 1 w. (in Demetriou et al. 2024); • Limassol, Laneia, 34.825, 32.921, 12.V.2007, Anastasios Legakis leg., J. Demetriou det., coordinates inferred with 1 km uncertainty radius; specimen code in Prof. Legakis’ collection: 1978, ZMUA, 1w.

##### Diagnosis.

*Camponotus limbouros* differs from all the known Eastern Mediterranean *Camponotus* in the presence of a distinctly concave dorsal propodeum [slightly concave dorsal propodeum can be observed only in some specimens of *C. fallax* and *C. aethiops-oertzeni* complex (Karaman et al. 2011)]. Historically, the species was confused with *C. vitiosus*, from which it differs in the presence of deep median emargination in clypeus in major workers and gynes, head in major workers never truncate anteriorly; presence of long and erect setae on frons and the ventral surface of head; densely imbricate mesosoma; and propodeum with erect setae present only on posterior part of its dorsum. Based on the morphological characters i.e., pronounced caste dimorphism, medium-sized body, subrectangular head with slightly convex lateral sides (in frontal view), arched mesosoma, anterior margin of clypeus regularly arched and lacking protrusion (with median emargination in major workers and gynes, we assign this species to the *Myrmentoma* subgenus. Within this subgenus, *C. limbouros* differs from the members of the *kiesenwetteri* species group in having a shiny and imbricate body sculpture; from members of the *lateralis* species group in having a regularly arched mesosoma in lateral view (metanotal groove absent) and the lack of saddle-shaped propodeum; from *Camponotus fallax* in the presence of erect setae on genae; from *Camponotus tergestinus* in the lack of erect setae on antennal scapus and posterior head. The species morphologically belongs to the *caryaea*/*fallax* species group [sensu Emery (1925) and Radchenko (1997)]. However, these species groups are not monophyletic based on molecular phylogenetic data and will need to be revised in the future (Blaimer et al. unpubl. data).

##### Description.

**Worker**. Measurements (*n* = 8). HL: 1.14–1.25 (1.21); HW: 0.93–1.1 (1.02); SL: 1.1–1.23 (1.16); EL: 0.37–0.41 (0.39); PW: 0.83–0.91 (0.86); PTH: 0.38–0.46 (0.43); PTW: 0.19–0.26 (0.22); ML: 1.61–1.82 (1.7); HFL: 1.2–1.3 (1.24); CI: 81–88 (84); SI: 108–122 (114); PI: 165–226 (195); FI: 69–76 (73) (Table S4). ***Color***. Head bright brown to brown, sometimes with gena and clypeus entirely or partially yellow; mandibles and antennal scapes yellow to yellowish-brown, sometimes funiculus slightly darker than scape. Mesosoma uniformly colored, slightly brighter than head or in the same shade; sometimes pronotum or promesonotum on dorsal and/or lateral sides with yellowish-brown to yellow patches that can cover almost the whole surface. Gaster black, sometimes with indistinct yellow-brown patch on the anterior slope of the first gastral tergite and with transparent and yellowish posterior margins of subsequent tergites. Legs uniformly colored brighter than mesosoma or in the same shade. If legs brighter than mesosoma then coxa yellowish-brown to brown, trochanters yellow, femora brown except yellow knee, tibiae yellowish-brown in the middle and gradually paler apically, tarsi yellow (Figs 2, 3). ***Head***. Subrectangular, sides below eyes softly rounded and softly converging anterad, above eyes regularly rounded, posterior margin convex (Fig. 3). Anterior clypeal margin convex, without median emargination. Clypeus densely imbricate but shiny, covered with short and sparse appressed pubescence and up to ten long and erect setae. Eyes large and oval. Frontal carinae short, extending to 1/3 the length of the head, regularly arched, antennal fossa shallow, imbricated. Frons densely imbricate but shiny, with thin median sulcus, short and sparse appressed pubescence, and with a row of up to 14 long, erect setae along the frontal carinae. Rest of the head surface distinctly imbricate from shiny to slightly opalescent, covered with short and sparse appressed pubescence. Gena with several short erect setae, head dorsum immediately posterior to frontal carinae and anterior to vertex each with a pair of long erect setae between these pairs four additional very short erect setae (Fig. 3). Antennal scape long; in frontal view almost straight, apex slightly widened; funiculus longer than scape, pedicel elongated (Fig. 3). Scape densely imbricate, slightly dull, covered with short and sparse appressed pubescence, without decumbent or erect setae. Mandibles short, rounded, imbricate but shiny, apical ^1^/_4_ of its length with longitudinal grooves, surface with fewer than ten moderately long, yellow setae. ***Mesosoma***. Moderately elongate, without metanotal groove. Promesonotum, in lateral view, regularly arched, propodeal dorsum shallowly concave (Fig. 2). Pronotal sides in dorsal view regularly convex (Fig. 2). Mesosoma densely imbricate; background shiny, covered with sparse and short appressed pubescence, on the lateral sides of the pronotum and mesonotum pubescence hardly visible. Promesonotum with several short, yellow erect setae, propodeum in posterior half with several long, yellow erect setae, slightly longer than the setae on the promesonotum. ***Petiole***. Squamiform, thin, in lateral view ~ 2.1× as high as wide, anterior face distinctly convex, posterior face almost flat, apex on sides regularly rounded, centrally truncate, apicolateral corners each with several long, erect setae. (Fig. 2). Petiole distinctly imbricate, shiny. ***Gaster***. With distinct but sparse imbrication, shiny. Whole gaster with short and sparse, indistinct appressed pubescence; dorsum of the first tergite with several long, yellow erect setae, posterior margin with a row of slightly shorter setae, transparent posterior margin covered with short appressed setae not extending to the posterior margin of tergite; tergites 2 and 3 anteriorly and posteriorly with a row of several long yellow setae (Fig. 2). ***Legs***. Imbricate. Femora and tibia distinctly flattened. Hind femora distinctly shorter than the mesosoma. Dorsal and lateral surfaces of femora and tibiae covered with very sparse, yellow and appressed pubescence, standing hairs absent. Ventral surfaces of hind tibiae with two or three erect spiniform setae.

**Major workers**. Measurements (*n* = 3). HL: 1.35–1.66 (1.48); HW: 1.21–1.57 (1.34); SL: 1.21–1.25 (1.23); EL: 0.41–0.43 (0.42); PW: 0.96–1.11 (1.02); PTH: 0.46–0.63 (0.53); PTW: 0.23–0.25 (0.24); ML: 1.81–2.14 (1.94); HFL: 1.36–1.46 (1.41); CI: 87–95 (91); SI: 80–102 (93); PI: 184–274 (219); FI: 68–75 (72) (Table S4). ***Color***. Whole body brownish-black to black. Legs and antennae ochraceous-brown. Coxa and femora apically yellowish-black. Pronotum brownish black (Fig. 4). ***Head***. Subrectangular, sides below eyes straight, above eyes slightly rounded apically, posterior margin convex (Fig. 5). Anterior clypeal margin convex, with median emargination. Gena with numerous short erect setae (Fig. 5). Remaining characters the same as in workers. ***Mesosoma***. As in workers. ***Petiole***. As in workers. ***Gaster***. Densely imbricate, shiny. Remaining characters as in workers. ***Legs***. As in workers.

**Gyne. *Measurements*** (*n* = 2). HL: 1.83–1.88; HW: 1.63–1.63; SL: 1.27–1.40; EL: 0.49–0.60; MSW: 1.36–1.43; PTH: 0.75–0.67; PTW: 0.34–0.29; ML: 2.81–2.80; HFL: 1.54–1.60; CI: 87–89; SI: 78–86; PI: 221–231; FI: 55–57 (Table S4).

***Color***. Head, mesosoma and gaster predominantly brown to black with yellow to brownish-black patches on first and second gastral tergite, pronotum, and laterobasal margins of scutum; petiolar squama entirely brownish-black or brown with yellowish lateral margins; coxa brownish-black to brown at base and yellow posteriorly, trochanters yellow, femora yellowish-brown to brown, apex of fore femora with a yellow patch, tibiae pale brown with yellowish apex, tarsi yellowish-brown; antennae ochraceous-yellow (Fig. 6). ***Head***. Subrectangular, sides straight, very softly converging anterad, above eyes regularly rounded, posterior margin slightly convex (Fig. 6). Anterior clypeal margin convex, with median emargination, with several long and short yellow setae. Clypeus densely imbricate and shiny, covered with short and sparse appressed pubescence and numerous erect setae grouping mostly on the sides and base of the clypeal plate. Eyes large and oval. Frontal carinae short, extending to half the length of the head, angularly arched, antennal fossa shallow, without special sculpture. Frons densely imbricate and shiny, with thin median sulcus, short and sparse appressed pubescence, and with a row of several moderately long, erect setae along frontal carinae and few additional short setae close to median sulcus. Rest of the head surface distinctly imbricate, shiny to slightly opalescent, covered with short and sparse appressed pubescence. Gena with numerous short erect setae, head dorsum immediately posterior to frontal carinae and anterior to vertex each with a pair of long erect setae, the ocellar area with two pairs each of long and short erect setae. Antennal scape moderately long; in frontal view almost straight, apex gradually widened; funiculus longer than the scape (Fig. 6). Scape densely imbricate, slightly dull, covered with short and sparse appressed pubescence, with 2–3 short erect setae. Mandibles short, rounded, strongly imbricate and shiny, in their basal part with setose punctures, apical half with longitudinal grooves, surface with fewer than 16 moderately long, yellow setae. ***Mesosoma***. Moderately long. Pronotum elongate, visible from above, imbricate, shiny, covered with short and sparse appressed hairs and basally with several long erect setae. Scutum approximately as long as wide, regularly convex (Fig. 6); densely imbricate and with small, sparse punctures and long basal grooves extending to half the length of the scutum, shiny, covered with sparse and short appressed pubescence and on each side with two pairs of long erect setae. Scutellum almost regularly semicircular; imbricate and shiny and with a few sparse punctures and several long erect setae. An- and katepisternum distinctly imbricated, shiny and with short and sparse pubescence. Propodeum convex, subangulate in profile, imbricate, posterolateral corners with several long erect setae (Fig. 6). ***Petiole***. Squamiform, thin, anterior face distinctly convex, posterior face almost flat, apex on the sides regularly rounded, centrally truncate, apicolateral corners with three long, erect setae. Petiole with distinct microstriae, shiny. ***Gaster***. Imbricate and shiny; with short and sparse, hardly visible appressed pubescence; top of the dorsum and close to the posterior margin of the first tergite with rows of several long, yellow erect setae, transparent posterior margin covered with short appressed setae not extending to the posterior margin of the tergite; tergites 2 and 3 anteriorly and close to posterior margin with a row of several yellow setae (Fig. 6). Each of gastral sternites with several long, yellow, and erect setae. ***Legs***. Stout. Dorsal and lateral surfaces of femora and tibiae covered with very sparse, yellow appressed pubescence and a few subdecumbent hairs. Posteroventral face of fore femora with several erect setae and anteroventral part of hind femora with three erect setae. Ventral surfaces of hind tibiae with two or three erect spiniform setae.

##### Biological notes.

**Figure 7.**
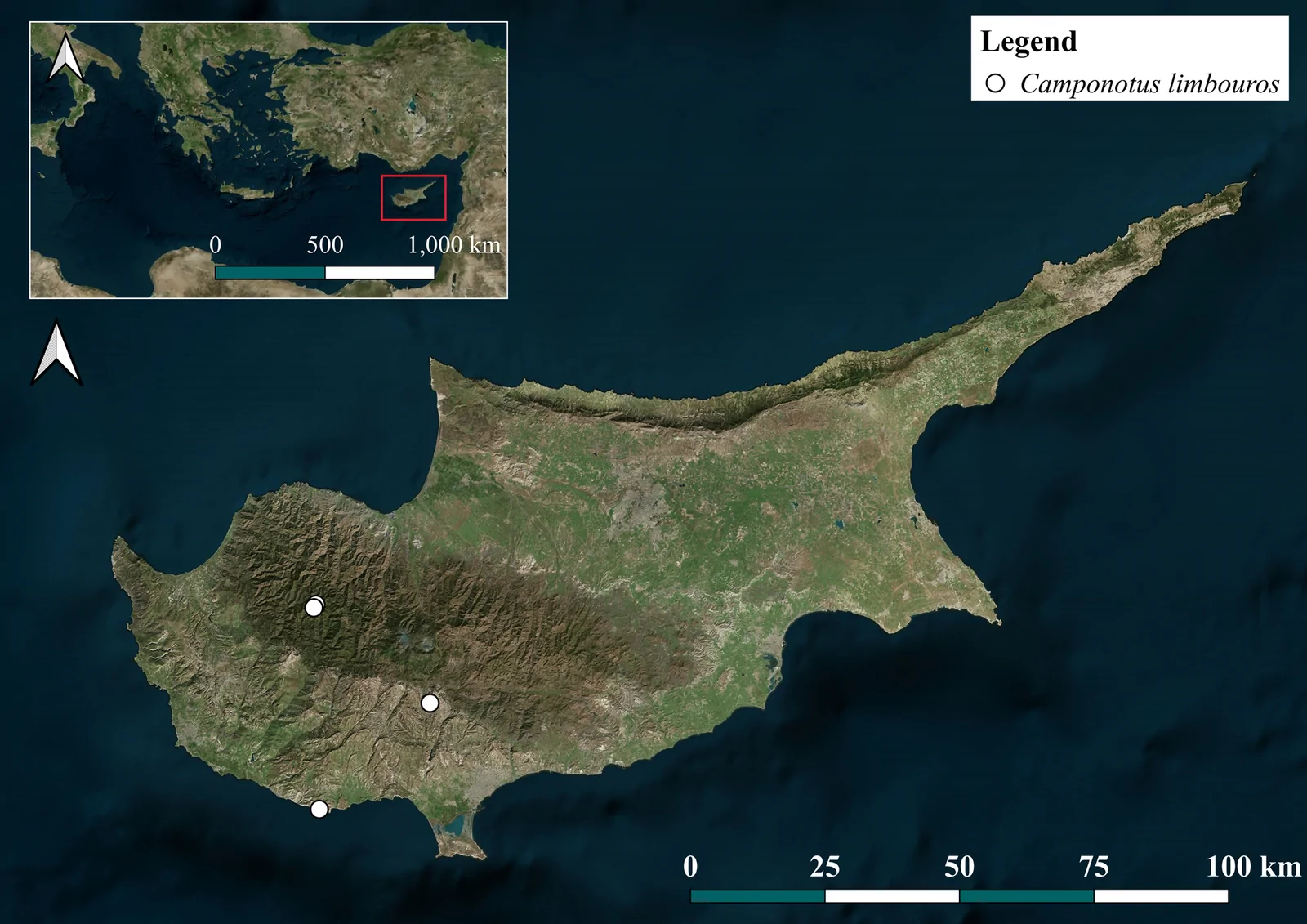
Distribution map of *Camponotus limbouros* sp. nov.

Our knowledge of the ecology of *C. limbouros* sp. nov. is still limited (Fig. 7). Specimens were collected in 2012 and 2024 in mixed cedar and pine forest in the famous refuge of Cypriot cedar *Cedrus brevifolia* (Hook. f.) A. Henry named Cedar Valley, at altitudes of 1130 and 1196 meters. Although no specimens were retrieved during an investigation in 2022, in 2024 a nest series was collected inside dry twigs on living trees of the endemic golden oak *Quercus alnifolia* Poech. The detected colony was small (fewer than 20 workers) and monogynous. The lone specimen collected with pitfall traps in 2013 (Pissouri, Trachonas) was found in phrygana with *Ceratonia siliqua*, while in 2007 Prof. (Emeritus) A. Legakis collected one specimen from Laneia village, with no additional notes on habitats or coordinates. Although habitat requirements are hard to interpret, the species is most probably arboreal, nesting inside twigs of standing trees; however, more research on its ecology and foraging patterns is necessary to shed light on any potential associations with specific tree species (e.g., *Q. alnifolia*).

##### Etymology.

The name derives from the word *λίμπουρος* or *λύμπουρος*, being the vernacular name of large ants in the Cypriot dialect. It derives from the adjective “lipouros/λίπουρος” meaning “the one missing a tail” i.e., *λιπ*(*ο*)-* (missing) + *οὐρά* (tail).

## Discussion

This article sheds light to the biodiversity and status of Cypriot *Camponotus*, elevating their number of endemic species to two, including *C. limbouros* sp. nov. and *C. troodosensis* Salata, Demetriou, Georgiadis & Borowiec, 2023 (Salata et al. 2023). Additionally, it elevates the total number of endemic species of ants in Cyprus to 19 while consequently reducing the number of non-native ant species to 17 by removing *C. cf. vitiosus* from the list of non-native ants in Cyprus (Demetriou et al. 2023b). This initial mistake was due to the collection originally of only a single gyne and minor worker of the new species, as well as the report of *C. cf. vitiosus* from neighboring Israel (Ionescu-Hirsch 2010 [2009]). This country has been identified as the source of *Allomerus auropunctatus* (Roger, 1863) in Cyprus (Loiseau et al. 2025; Longino and Branstetter 2026), thereby strengthening the original hypothesis. Only collecting the full nest sample and obtaining COI data revealed that the Cypriot populations represent a distinct and endemic species with unique morphological characters.

The ecology of *C. limbouros* sp. nov. is largely unknown, necessitating further research on its distribution, foraging habits, associations, threats and conservation status. The golden oak *Quercus alnifolia*, an endemic and the national tree of Cyprus, is protected by the Forest Law and Habitats Directive 92/43/EEC [“Scrub and low forest vegetation with *Quercus alnifolia*” (9390)] and is shown as a suitable nesting host for the species (Cedar Valley). Nevertheless, other localities known for the species host both *Q. infectoria veneris* and *Q. coccifera calliprinos* (i.e., Pissouri and Lania). As such, *C. limbouros* sp. nov. may potentially utilize various *Quercus* spp. as nesting sites or potentially other unrelated plants such as *C. siliqua* (prominent in the sampling site of Pissouri). Given its arboreal lifestyle the species may be threatened by forest fires and/or land-use changes affecting the quality and extent of its habitats and tree associates. Nevertheless, these hypotheses should be addressed through additional surveys.

DNA barcoding is highlighted as an important tool facilitating efforts towards identifying and resolving the taxonomic status of species-level taxa, including that of the species described herein. The COI barcode data clearly indicate the genetic distinctness of *C. limbouros* sp. nov. and, together with the morphological evidence, support its recognition as a new species. Although its precise phylogenetic position will require further investigation using nuclear genetic markers, it is likely that this species belongs to the subgenus *Myrmentoma* and we have therefore assigned the species to this subgenus. We acknowledge that the subgeneric classification of *Camponotus* is at least in part artificial and does not reflect phylogeny (Ward et al. 2025). Ongoing unpublished phylogenomic work of some of the authors, however, shows that most species assigned to *Myrmentoma* form a clade and that this subgenus will eventually be phylogenetically validated. For consistency, we chose to report subgeneric assignments for all species included in the COI tree. However, our COI gene tree should not be mistaken for a phylogeny and does not lend sufficient genetic support to shed light on the subgeneric classification of *Camponotus* as a whole, which is the reason we have refrained from commenting further on our phylogenetic results.

Lastly, an important next step will be the development of a comprehensive DNA barcode reference library for the Cypriot myrmecofauna, thereby complementing and expanding existing European databases (Menchetti et al. 2026). Future applications of such a reference framework could, for example, include eDNA- or metabarcoding-based approaches for monitoring the distribution and habitat associations of both native and non-native ant species in Cyprus.

## Supplementary Material

XML Treatment for Camponotus limbouros

## Additional information

### Artificial Intelligence (AI) use

The authors accept full responsibility for the content of the manuscript, including the disclosure of any use of AI.

No AI tools were used in the preparation of this manuscript.

### Author ORCIDs

J. Demetriou https://orcid.org/0000-0001-5273-7109

B.B. Blaimer https://orcid.org/0000-0002-8961-9998

C. Georgiadis https://orcid.org/0000-0002-2728-3122

J.H. Voges https://orcid.org/0009-0007-9147-4533

B. Guénard https://orcid.org/0000-0002-7144-1175

L. Borowiec https://orcid.org/0000-0001-5668-6855

S. Salata https://orcid.org/0000-0003-0811-2309

### Data availability

All of the data that support the findings of this study are available in the main text or Supplementary Information.

## Supplementary materials

Supplementary material 1 Cytochrome oxidase I (COI) tree for Mediterranean *Camponotus* species Data type pdf Explanation note Extended cytochrome oxidase I (COI) tree comprising all Camponotus (Myrmentoma) species for which sequence data were available. https://binary.pensoft.net/file/1802903This dataset is made available under the Open Database License (http://opendatacommons.org/licenses/odbl/1.0/). The Open Database License (ODbL) is a license agreement intended to allow users to freely share, modify, and use this Dataset while maintaining this same freedom for others, provided that the original source and author(s) are credited.Author: Bonnie B. Blaimer

Supplementary material 2 List of sequences downloaded from BOLD. Data shown exactly as downloaded, except for GenusSpecies field Data type xlsx Explanation note List of sequences downloaded from BOLD. Data shown exactly as downloaded, except for GenusSpecies field. https://binary.pensoft.net/file/1802904This dataset is made available under the Open Database License (http://opendatacommons.org/licenses/odbl/1.0/). The Open Database License (ODbL) is a license agreement intended to allow users to freely share, modify, and use this Dataset while maintaining this same freedom for others, provided that the original source and author(s) are credited.Author: Bonnie B. Blaimer

Supplementary material 3 Data for newly generated sequences for this study Data type xlsx Explanation note Shown in same format as table SS1. https://binary.pensoft.net/file/1802905This dataset is made available under the Open Database License (http://opendatacommons.org/licenses/odbl/1.0/). The Open Database License (ODbL) is a license agreement intended to allow users to freely share, modify, and use this Dataset while maintaining this same freedom for others, provided that the original source and author(s) are credited.Author: Bonnie B. Blaimer

Supplementary material 4 Estimates of sequence divergence Data type xlsx Explanation note Analyses were conducted using the Kimura 2-parameter model (Kimura 1980) in MEGA12 (Stecher et al. 2020 & 2025). https://binary.pensoft.net/file/1802906This dataset is made available under the Open Database License (http://opendatacommons.org/licenses/odbl/1.0/). The Open Database License (ODbL) is a license agreement intended to allow users to freely share, modify, and use this Dataset while maintaining this same freedom for others, provided that the original source and author(s) are credited.Author: Bonnie B. Blaimer

Supplementary material 5 Detailed measurements of examined specimens of *Camponotus limbouros* sp. nov. Data type xlsx https://binary.pensoft.net/file/1802907This dataset is made available under the Open Database License (http://opendatacommons.org/licenses/odbl/1.0/). The Open Database License (ODbL) is a license agreement intended to allow users to freely share, modify, and use this Dataset while maintaining this same freedom for others, provided that the original source and author(s) are credited. Sebastian Salata, Lech Borowiec
