# Supplementary material for: Losing a non-native, gaining an endemic: Description of Camponotus (Myrmentoma) limbouros sp. nov. (Hymenoptera, Formicidae) from Cyprus
Source: Zookeys. 2026 Oct 6;1294:151–74. doi: 10.3897/zookeys.1294.205360 (PMC13662622; doi:10.3897/zookeys.1294.205360)
Supplement: Supplementary file 1 [file zookeys-1294-151_article-205360__-s001.pdf]

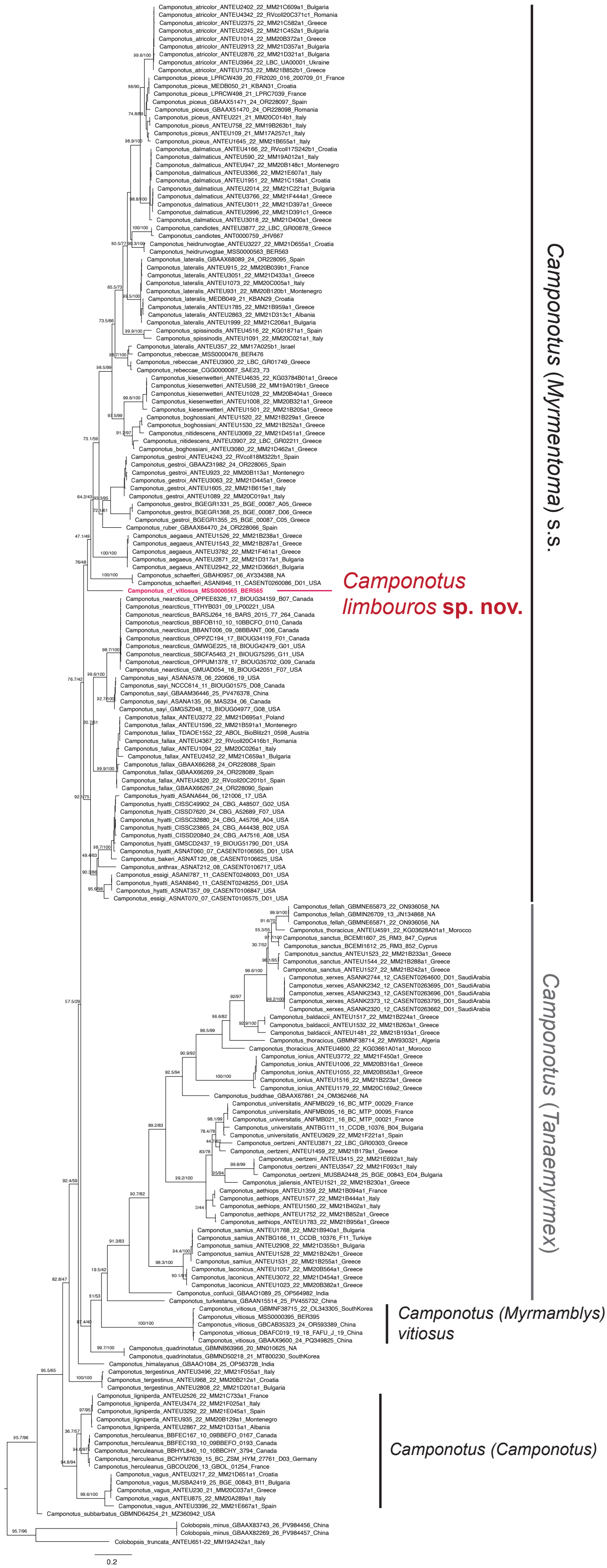

Camponotus (Myrmentoma) s.s.
Camponotus_cf_vitiosus_MSS0000565_BER565
Camponotus limbouros sp. nov.
Camponotus (Tanaemyrmex)
Camponotus (Myrmamblys) vitiosus
Camponotus (Camponotus)
0.2
